# Evolutionary engineering improves tolerance for medium-chain alcohols in *Saccharomyces cerevisiae*

**DOI:** 10.1186/s13068-018-1089-9

**Published:** 2018-04-02

**Authors:** Stephanie A. Davis López, Douglas Andrew Griffith, Brian Choi, Jamie H. D. Cate, Danielle Tullman-Ercek

**Affiliations:** 1 Next Interactions, Richmond, CA 94806 USA; 2 Department of Chemical and Biological Engineering, Northwestern University, 2145 Sheridan Road, Tech E-136, Evanston, IL 60208-3109 USA; 3 Department of Chemical and Biomolecular Engineering, University of California, Berkeley, CA 94720 USA; 4 Department of Chemistry, University of California, Berkeley, CA 94720 USA

**Keywords:** *Saccharomyces cerevisiae*, Medium-chain alcohols, Biofuels, Translation initiation, eIF2, eIF2B, Alcohol tolerance

## Abstract

**Background:**

Yeast-based chemical production is an environmentally friendly alternative to petroleum-based production or processes that involve harsh chemicals. However, many potential alcohol biofuels, such as *n*-butanol, isobutanol and *n*-hexanol, are toxic to production organisms, lowering the efficiency and cost-effectiveness of these processes. We set out to improve the tolerance of *Saccharomyces cerevisiae* toward these alcohols.

**Results:**

We evolved the laboratory strain of *S. cerevisiae* BY4741 to be more tolerant toward *n*-hexanol and show that the mutations which confer tolerance occur in proteins of the translation initiation complex. We found that *n*-hexanol inhibits initiation of translation and evolved mutations in the α subunit of eIF2 and the γ subunit of its guanine exchange factor eIF2B rescue this inhibition. We further demonstrate that translation initiation is affected by other alcohols such as *n*-pentanol and *n*-heptanol, and that mutations in the eIF2 and eIF2B complexes greatly improve tolerance to these medium-chain alcohols.

**Conclusions:**

We successfully generated *S. cerevisiae* strains that have improved tolerance toward medium-chain alcohols and have demonstrated that the causative mutations overcome inhibition of translation initiation by these alcohols.

**Electronic supplementary material:**

The online version of this article (10.1186/s13068-018-1089-9) contains supplementary material, which is available to authorized users.

## Background

The brewer’s yeast *Saccharomyces cerevisiae* is the production organism of choice for the most prominent commercial biofuel, bioethanol. In recent years, there has been growing interest in using this yeast for the production of advanced higher chain alcohol biofuels, such as *n*-butanol [1–6] and isobutanol [7, 8], and for bio-alcohols with other uses such as the prenyl alcohols, farnesol, nerolidol, and geranylgeraniol, which are fragrance components of essential oils [9]. A major challenge when using *S. cerevisiae* to produce bio-alcohols, however, is that many of these compounds are toxic to cells. Ethanol and other alcohols accumulate in the yeast plasma membrane increasing its fluidity, and altering its stability and structure [10, 11]. They severely perturb membrane function by increasing the proton permeability of the plasma membrane, which diminishes the proton-motive force available to drive secondary active solute and nutrient transporters [10–12]. In addition to this indirect effect on membrane protein function, ethanol inhibits water transport across the plasma membrane by a direct interaction with the aquaporin *AQY1* [10], and higher chained *n*-alcohols (from propanol to hexanol) are inhibitors of the multidrug-resistance pumps Pdr5p and Snq2p [13]. The increased membrane fluidity caused by alcohols also results in a loss of intracellular molecules such as ATP, RNA, proteins and phospholipids [12, 13]. Taken together, the various effects of alcohols on yeast cells lead to the impairment of essential physiological processes including cellular energy maintenance, growth inhibition, and even cell death. Understanding these effects is the next step toward engineering solutions to improve tolerance.

Fusel alcohols, such as *n*-butanol and isoamyl alcohol, are by-products of amino acid metabolism in *S. cerevisiae,* and a promising series of studies revealed that these alcohols inhibit translation initiation by targeting the eIF2B guanine nucleotide exchange factor (GEF) that recycles the eIF2 complex from a GDP- to a GTP-bound form that is competent for translation initiation [14, 15]. eIF2 plays an important role in translation initiation as it brings the methionine-charged initiator tRNA to the 40S subunit of the ribosome [16, 17]. To explain the inhibition by fusel alcohols, Taylor et al. [14] suggested that the alcohols either bind directly to eIF2B, or alter as-yet unidentified posttranslational modifications, such as phosphorylation, which are critical to the translation initiation process [15]. Moreover, mutations in eIF2B can modulate this inhibition [14, 15].

Adaptive laboratory evolution (ALE) is increasingly used as a technique for untargeted strain optimization [18, 19] and was successfully employed to identify biological solutions to biofuel and alcohol toxicity in *S. cerevisiae* [11, 20–22]. Here we used ALE to identify genetic targets that can be exploited to alleviate medium-chain alcohol toxicity in *S. cerevisiae*. We subjected wild-type yeast to an ALE experiment by gradually increasing the *n*-hexanol concentration in the growth medium to enrich for *n*-hexanol-tolerant *S. cerevisiae* strains. After two 30-day rounds of evolution, we identified strains with significantly more *n*-hexanol tolerance. These evolved strains also showed greater tolerance to a wide range other medium-chain alcohols (from propanol to octanol). Genome sequencing of our tolerant strains revealed three interesting mutations in proteins crucial for translation initiation. We found a D77Y mutation in the eIF2α subunit, Sui2p, a D85E mutation in the eIF2Bγ subunit, Gcd1p, and a R56C mutation in the eIF2Bβ subunit, Gcd7p. We further show that *n*-hexanol, like *n*-butanol and isoamyl alcohol [14, 15], inhibits translation initiation, and that individually reverse-engineering the D77Y mutation in eIF2α and the D85E mutation in eIF2Bγ confers tolerance to *n*-hexanol by relieving this inhibition. The D85E mutation in eIF2Bγ is the first mutation identified in this subunit that reduces sensitivity to alcohols, and the D77Y mutation in eIF2α is the first mutation in eIF2 shown to mitigate alcohol-dependent translational inhibition. This is the first report of tolerance engineering to *n*-hexanol in *S. cerevisiae*.

## Methods

### Yeast strains, media, and molecular biology for generation of yeast strains

*Saccharomyces cerevisiae* strains and plasmids used in this study are listed in Table 1. All adaptive evolution experiments were performed in the background of BY4741 (*MATa his3Δ1 leu2Δ0 met15Δ0 ura3Δ0*). Yeast strains were grown in YPD medium [1% yeast extract (Amresco), 2% bacto-peptone (BD Chemical), 2% dextrose (Amresco)] unless otherwise indicated.

**Table 1:** List of *Saccharomyces cerevisiae* strains and plasmids used in this study and their genotypes

Strain	Genotype	Plasmid	Plasmid description	Source
BY4741	*MATa his3Δ1 leu2Δ0 met15Δ0 ura3Δ0*	None		John Dueber^a^
sSD003	*MATa his3Δ1 leu2Δ0 met15Δ0 ura3Δ0* ***gcd1*** **-** ***1 cit2*** **-** ***1***	None		This study
sSD006	*MATa his3Δ1 leu2Δ0 met15Δ0 ura3Δ0* ***gcd7*** **-** ***2***	None		This study
sSD019	*MATa his3Δ1 leu2Δ0 met15Δ0 ura3Δ0* ***gcd1*** **-** ***1 cit2*** **-** ***1 pdr5*** **-** ***1 ubp13*** **-** ***1 lsb6*** **-** ***1 nst1*** **-** ***1 cox1*** **-** ***1***	None		This study
sSD021	*MATa his3Δ1 leu2Δ0 met15Δ0 ura3Δ0* ***gcd7*** **-** ***2 sui2*** **-** ***2 pdr5*** **-** ***2 sey1*** **-** ***2***	None		This study
sSD029	*MATa his3Δ1 leu2Δ0 met15Δ0 ura3Δ0 gcd1Δ::KanMX4*	pSD450	pAH056 *CEN6/ARS4 URA3 gcd1*-*1*	This study
sSD040	*MATa his3Δ1 leu2Δ0 met15Δ0 ura3Δ0 gcd7Δ::His3*	pSD469	pAH005 *CEN6/ARS4 LEU2 gcd7*-*2*	This study
sSD053	*MATa his3Δ1 leu2Δ0 met15Δ0 ura3Δ0 gcd1Δ::KanMX4*	pSD442	pAH056 *CEN6/ARS4 URA3 GCD1*	This study
sSD054	*MATa his3Δ1 leu2Δ0 met15Δ0 ura3Δ0 gcd7Δ::His3*	pSD474	pAH005 *CEN6/ARS4 LEU2 GCD7*	This study
sSD055	*MATa his3Δ1 leu2Δ0 met15Δ0 ura3Δ0 sui2Δ::KanMX4*	pSD444	pAH056 *CEN6/ARS4 URA3 SUI2*	This study
sSD056	*MATa his3Δ1 leu2Δ0 met15Δ0 ura3Δ0 sui2Δ::KanMX4*	pSD452	pAH056 *CEN6/ARS4 URA3 sui2*-*2*	This study
sSD058	*MATa his3Δ1 leu2Δ0 met15Δ0 ura3Δ0 sui2Δ::KanMX4 gcd7Δ::His3*	pSD452/pSD469	pAH056 *CEN6/ARS4 URA3 sui2*-*2* pAH005 *CEN6/ARS4 LEU2 gcd7*-*2*	This study
sSD060	*MATa his3Δ1 leu2Δ0 met15Δ0 ura3Δ0 sui2Δ::KanMX4 gcd7Δ::His3*	pSD444/pSD474	pAH056 *CEN6/ARS4 URA3 SUI2* pAH005 *CEN6/ARS4 LEU2 GCD7*	This study
sSD061	*MATa his3Δ1 leu2Δ0 met15Δ0 ura3Δ0*	pSD442	pAH056 *CEN6/ARS4 URA3 GCD1*	This study
sSD062	*MATa his3Δ1 leu2Δ0 met15Δ0 ura3Δ0*	pSD450	pAH056 *CEN6/ARS4 URA3 gcd1*-*1*	This study
sSD063	*MATa his3Δ1 leu2Δ0 met15Δ0 ura3Δ0*	pSD444	pAH056 *CEN6/ARS4 URA3 SUI2*	This study
sSD064	*MATa his3Δ1 leu2Δ0 met15Δ0 ura3Δ0*	pSD452	pAH056 *CEN6/ARS4 URA3 sui2*-*2*	This study

Highlighted in bold are mutant alleles identified in the evolved strains

^a^Dept. of Bioengineering, University of California Berkeley

Plasmid construction steps were performed using standard methods for recombinant DNA work [23] in *E. coli* strain DH10B and by in vivo homologous recombination (HR) in BY4741 [24]. BY4741 was transformed to *URA*^+^, *LEU*^+^ and *HIS*^+^ by a whole cell high-efficiency lithium acetate method [25]. To reverse engineer the mutant alleles *gcd1*-*1*, *gcd7*-*2*, and *sui2*-*2,* these genes were cloned individually by HR onto low-copy CEN6/ARS4 plasmids, and then individually or in combination transformed into BY4741 (see Table 1). Constructs and strains for the wild-type alleles were generated to serve as controls. The wild-type genes in the genome of transformed strains matching those on the plasmids were then deleted. To construct the plasmids, genomic DNA (gDNA) was isolated from strains sSD019 and sSD0021 with the YeaStar™ genomic DNA kit (Zymo Research, Irvine, CA), and used as a template to amplify *gcd1*-*1*, *gcd7*-*2*, and *sui2*-*2* by PCR. Wild-type alleles for these genes were amplified from gDNA extracted from BY4741. All primer sequences used in this study can be found in Additional file 1: Table S1. The PCR primers added 38 bp of sequence to the ends of the genes that are homologous to those flanking the *Spe*I and *Bam*HI sites at the desired point of insertion in the destination vectors. Alleles *gcd7*-*2* and *GCD7*^+^ were cloned into *Spe*I/*Bam*HI-digested pAH005, which carries the *LEU2* marker [26], to generate pSD469 and pSD474, respectively, and *gcd1*-*1*, *sui2*-*2*, *GCD1*^+^, and *SUI2*^+^ were cloned into *Spe*I/*Bam*HI-digested pAH056, which carries the *URA3* marker [26] to create pSD450, pSD452, pSD442 and pSD444, respectively. Transformants were selected on solid synthetic dropout (SD) media (containing 20 g/l agar) lacking uracil or leucine, or both (0.67% yeast nitrogen base (BD Chemical), 2% dextrose, 0.2% amino acid mix without uracil or leucine or both (US Biological). Recombinant plasmids were isolated using the QIAprep Spin Miniprep kit (Qiagen) [27], shuttled into *E. coli*, and the integrity of cloned sequences was confirmed by dye-terminator sequencing.

Gene deletions in strains carrying plasmids for wild-type and mutant alleles were performed using a cloning-free PCR-based allele replacement method [28]. The *HIS3* selection marker from plasmid pML840 [26] was PCR amplified to give a product with sequences homologous (~ 40 bp) to those flanking the *GCD7* gene. Similarly, PCR products with the kanamycin/G418 resistance (KanMX) marker from BY4741 ∆tpo1 (*MATa his3∆1 leu2∆0 met15∆0 ura3∆0 tpo1∆::kanMX4*) were generated to delete *GCD1* and *SUI2.* Strains BY4741[pSD469] and BY4741[pSD474] were transformed with the *GCD7* deletion cassette to obtain sSD040 and sSD050, respectively. *LEU*^+^ *HIS*^+^ transformants were selected on SD medium lacking leucine and histidine. Strains BY4741[pSD450] and BY4741[pSD442] were transformed with the *GCD1* cassette to obtain sSD029 and sSD053, respectively, and strains BY4741[pSD444] and BY4741[pSD452] were transformed with the *SUI2* cassette to obtain sSD055 and sSD055, respectively. *URA*^+^ *KanMX*^+^ transformants were selected on SD medium lacking uracil and supplemented with 200 mg/l Geneticin (G418 sulfate). Finally, BY4741[pSD452/pSD469] and BY4741[pSD444/pSD474] were transformed with the *GCD7* and *SUI2* deletion cassettes to obtain sSD058 and sSD060, respectively. These transformations were plated on SD medium lacking uracil, leucine and histidine, and uracil, leucine with Geneticin, respectively. Gene deletions were confirmed by diagnostic PCR across the junctions of the expected integration site on genomic DNA templates isolated from these strains. Primer pairs were GCD1-F/KanMX-diagnostic-R and GCD1-R/KanMX-diagnostic-F, for the *GCD1* gene deletion, SUI2-F/KanMX-diagnostic-R and SUI2-R/KanMX-diagnostic-F, for the *SUI2* deletion, and GCD7-HIS3-F/HIS3-diagnostic-R for *HIS3*, for the *GCD7* deletion (Additional file 1: Table S1).

Single amino-acid substitution libraries at positions D85 in Gcd1p and D77 Sui2p D77 were generated by PCR in which the codon for the substituted amino-acid was randomized by degenerate NNN and NNK primers (Additional file 1: Table S1), respectively, where N is an equimolar mixture of all four nucleotides and K is an equimolar mixture of bases G and T. *GCD1* and *SUI2* genes were amplified as two products using two partially overlapping mutagenic primers and two primers outside the coding sequence, and pSD442 and pSD444 plasmid DNA as templates, respectively. The mutant gene sequences were introduced into the appropriate destination vector by HR in yeast. The destination vectors for Gcd1p and Sui2p mutants were constructed by HR from pSD442 (*Cla*I digested) and pSD444 (*Bgl*II digested) by replacing the *GCD1* and *SUI2* coding sequence with the KanMX marker. For each library of single amino-acid substitution yeast mutants, 96 colonies were sequenced and analyzed.

### Adaptive laboratory evolution

ALE was performed by a serial batch transfer procedure and by batch fermentation in a chemostat in the presence of inhibitory concentrations of *n*-hexanol as a selective pressure. In the serial batch procedure, a single aerobic culture of strain BY4741 was initiated in 10 ml YPD supplemented with 0.12% (v/v) *n*-hexanol in 50 ml screw-cap tubes and grown at 30 °C and 200 rpm. This culture was passaged daily in fresh YPD with 0.12% *n*-hexanol. After 30 daily passages, a diluted aliquot of the final culture was plated for single colonies on YPD. Yeast populations derived from a number of colonies were tested for their *n*-hexanol tolerance (see “Tolerance testing” below), as compared to the parental strain, and the best performing strain was designated sSD003.

For batch fermentation, BY4741 was cultivated in a 0.5 l chemostat containing 0.5 l YPD. Fresh medium was fed at a constant flow-rate in the range 0.25–0.5 ml/min so that the OD_600_ of the culture was maintained in the range 0.5–2.0 AU. The initial *n*-hexanol concentration was 0.1%, but as the cell density increased, the *n*-hexanol concentration was steadily increased to 0.3%. The chemostat had no air inlet or outlet, so the culture was grown under microaerobic conditions, mimicking those found in large-scale fermentation tanks. The chemostat was kept in a 30 °C room and the culture mixed with a medium-speed stir bar. At the end of the 30 day fermentation, several strains derived from the final culture were tested for *n*-hexanol tolerance, and the best performing strain was designated sSD006.

Strains sSD003 and sSD006 were subjected individually to a second 30-day round of adaptive evolution by serial batch transfer in tubes. The starting *n*-hexanol concentration was 0.12%, and this was increased gradually to 0.2% over the 30-day period. After tolerance testing of several strains derived from sSD003, we identified strain sSD019 which showed significantly increased *n*-hexanol tolerance compared to the parental strain. Similarly, a significantly more *n*-hexanol-tolerant strain, sSD021, was obtained from the adaptive evolution of sSD006.

### Tolerance testing

Strains were evaluated for tolerance to medium-chain alcohols by determining growth curves. Overnight cultures were diluted to an OD_600_ of 0.3 AU in 5 ml YPD and then mixed with 5 ml YPD containing two times the desired final concentration of the test alcohol (expressed as v/v). Cultures were grown in closed 50 ml screw-cap tubes (to prevent the evaporation of test alcohols) at 30 °C and 280 rpm. The OD_600_ of the cultures was measured every 2 h in triplicate with a Genesys 20 spectrophotometer. The average specific growth rate (*μ*_avg_) for each culture was calculated for the time period between 2 and 10 h (except where otherwise indicated) using the following equation: *μ* = (ln*X*_2_ − ln*X*_1_)/(*t*_1_ − *t*_2_), where *X*_2_ and *X*_1_ are the cell density at time *t*_2_ and *t*_1_.

### Genomic sequencing

Genomic DNA was isolated from the evolved strains with the YeaStar™ genomic DNA kit. Barcode-indexed genomic DNA sequencing libraries were generated from 1 µg of DNA each. The DNA samples were sheared on a Diagenode Bioruptor NGS sonicator (Diagenode, Liege, Belgium) to an average length of 300 bp. The libraries were prepared with the TruSeq Library Prep Kit (Illumina, San Diego, CA) following the instructions of the manufacturer. Libraries were analyzed with a Bioanalyzer 2100 instrument (Agilent, Santa Clara, CA), quantified by fluorometry on a Qubit instrument (LifeTechnologies, Carlsbad, CA) and pooled in equimolar ratios. The library pool was quantified by qPCR with a Kapa Library Quant RT-qPCR kit (Kapa, Cape Town, South Africa). 8.2 pM of library was loaded and ran on 1 lane of an Illumina HiSeq 2000 (Illumina, San Diego, CA). The raw reads were trimmed to remove sequencing adaptors as well as quality trimmed using Trimmomatic v0.36 [29] to have a minimum quality of 14 in a sliding window of 4. Paired reads that are longer than 36 bp were kept for further analysis. Mapping to the *S. cerevisiae* S288C genome (R64-2-1) obtained from the Saccharomyces Genome Database was done using the Burrows Wheel Aligner v0.7.12 MEM algorithm [30]. The mapped reads were then sorted and duplicated reads were marked using Picard v2.9.2. Variant calling was done using freebayes v1.1.0.46 [31] with the ploidy option set to 1. Variants with quality less than 20 were then filtered out using vcflib v1.0.0. The variants found in parental strain were removed from those found in each evolved strain with bedtools v2.26.0 [32]. Annotation was done using both SnpEff v4.3 [33] and Bcftools v1.6. The SnpEff output was used to decipher the potential effects of the mutations. Raw reads have been deposited in the NCBI Sequence Read Archive under the accession number PRJNA417511.

### Polysome profiling

Polysome analysis was performed as previously described [34]. Briefly, strains were grown in 250 ml of YPD to an OD_600_ of ~ 1 AU. The test alcohol was then added and the culture incubated for a further 30 min. Cycloheximide (100 µg/ml) was added to each culture for 5 min to arrest translation. Cells were harvested, washed twice, and then lysed with glass beads in ice-cold breaking buffer (20 mM Tris/HCl pH 7.4, 50 mM KCl, 10 mM MgCl_2_, 1 mM DTT, 100 µg/ml cycloheximide, 50 U/ml SuperRNasein, and protease inhibitors), followed by centrifugation at 13,000×*g* for 20 min at 4 °C. 20 A_260 nm_ units (800 μg of RNA) of each supernatant was fractionated on 10–50% sucrose gradients for 3 h at 35,000 rpm and 4 °C using a SW40-Ti rotor in a Optima L-90 K ultracentrifuge (Beckman Coulter). Polysome profiles were obtained by monitoring the RNA absorbance at 254 nm along the gradient using a BR-188 Density Gradient Fractionation System, and recording the output from the UA-6 Detector with programmable RS-232 software (Brandel, Gaithersburg, Maryland). Polysome-to-monosome (P/M) ratios were determined by calculating the area under the curve corresponding to the polyribosome peaks (more than two ribosomes) divided by the area under the curve for the monosome (80S) peak.

## Results

### Adaptive laboratory evolution of medium-chain alcohol tolerance in *S. cerevisiae*

**Fig. 1:**
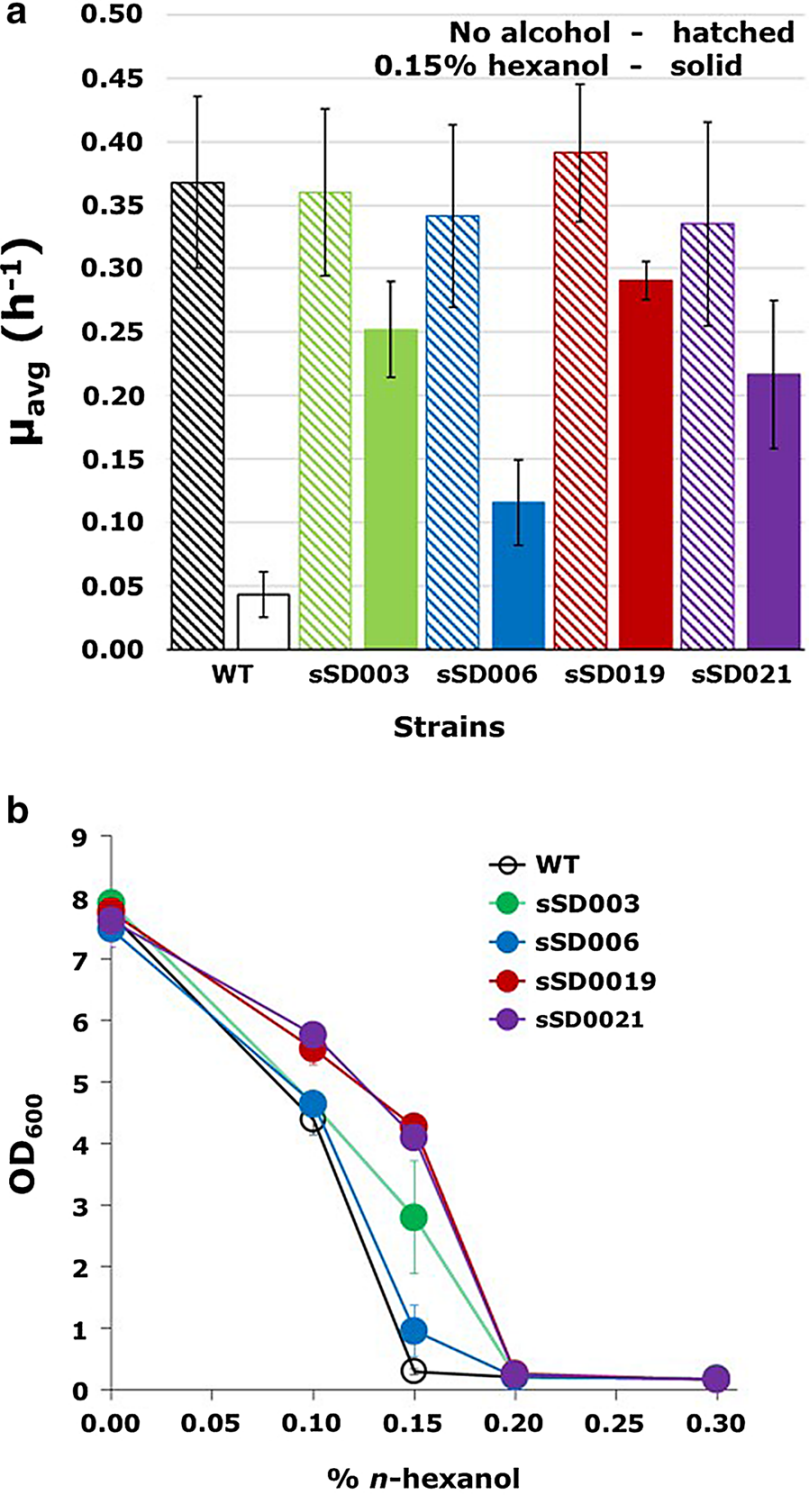
*n*-Hexanol tolerance of evolved strains. **a** Average specific growth rates (*µ*_avg_) for wild-type (WT) and evolved strains grown in 10 ml YPD + 0.15% *n*-hexanol in 50 ml culture tubes were calculated from growth curves as described in “Methods”. Values are mean ± standard deviation from 9, 7, 4, 4, and 4 independent cultures of WT, sSD003, sSD006, sSD019, sSD021, respectively. **b** Biomass yield (OD_600_) measured at 24 h for three independent cultures of WT and evolved strains in the presence of *n*-hexanol concentrations in the range 0–0.3%

To select for spontaneous mutants with greater tolerance to *n*-hexanol, two independent evolutionary lineages of wild-type BY4741 were established in the presence of the alcohol. The first of these was grown aerobically in 50 ml tubes in the presence of 0.12% *n*-hexanol and was serially passaged for 30 days (serial batch transfer). This concentration was chosen based on our observations that it resulted in severely limited growth. Several strains were then isolated from this population as single colonies on plates and tested for *n*-hexanol tolerance. Tolerance testing was performed by determining growth curves with measurements made every 2 h. Strains were grown in closed 50 ml screw-cap tubes to prevent evaporation of *n*-hexanol, and the average specific growth rates (*μ*_avg_) were calculated for the time period between 0 and 10 h to ensure that measurements were made on aerobically growing yeast (see representative growth curves for evolved strains in Additional file 1: Figure S1). For the best performing strain, sSD003, *µ*_avg_ was increased ~ sixfold compared to the parental strain (*P *< 0.001) in the presence of 0.15% *n*-hexanol (Fig. 1a). sSD003 was then subjected to another 30-day round of adaptive evolution by serial batch transfer with an *n*-hexanol concentration of 0.12% that was increased gradually to 0.21%. The best performing strain of those tested from the final culture was designated sSD019 and it grew ~ sevenfold faster than the wild-type strain (*P *< 0.001) in 0.15% *n*-hexanol (Fig. 1a).

The second evolutionary lineage was grown in a microaerobic chemostat for 30 days with an initial *n*-hexanol concentration of 0.1% that was increased steadily with increasing cell density to a final concentration of 0.3%. Strain sSD006 was isolated from the final culture that grew ~ threefold faster than the parental strain (*P *< 0.001). This lineage was further extended by 30 days of serial batch transfer in tubes with 0.12–0.21% *n*-hexanol. This allowed the isolation of sSD021 which exhibited a ~ 5-fold increased *µ*_avg_ compared to wild-type BY4741 (*P *< 0.001) (Fig. 1a). In the absence of *n*-hexanol, the evolved mutants grew as well as the wild-type strain [0.37 ± 0.07 h^−1^ (*n* = 21), 0.36 ± 0.07 h^−1^ (*n* = 18), 0.34 ± 0.07 h^−1^ (*n* = 12), 0.39 ± 0.05 h^−1^ (*n* = 15), and 0.34 ± 0.08 h^−1^ (*n* = 14) for WT, sSD003, sSD006, sSD019 and sSD021, respectively].

To identify the upper range of *n*-hexanol tolerance for the evolved strains, we measured the biomass (OD_600_) of cultures of the WT and evolved strains following 24 h growth in the presence of *n*-hexanol concentrations in the range 0–0.3% (Fig. 1b). At an *n*-hexanol concentration of 0.2%, the OD_600_ values for sSD003 and sSD006 were not significantly different from that for the WT strain [0.234 ± 0.013 (*n* = 3), 0.211 ± 0.009 (*n* = 3), and 0.202 ± 0.018 (*n* = 3), respectively]. In contrast, the OD_600_ values for the more evolved strains sSD019 and sSD021 were significantly greater than that for the WT strain (*P *< 0.05) [0.258 ± 0.023 (*n* = 3) and 0.243 ± 0.018 (*n* = 3), respectively], demonstrating that they can grow at this concentration of *n*-hexanol.

To investigate whether the evolved *n*-hexanol-tolerant strains were also more tolerant to other higher alcohols, we tested their growth in the presence of toxic concentrations of *n*-propanol (PrOH), *n*-butanol (BuOH), isobutanol (iBuOH), amyl alcohol (AOH), isoamyl alcohol (iAOH), *n*-pentanol (PtOH), *n*-heptanol (HpOH), and 3-octanol (OcOH) (Table 2). All alcohols tested inhibited the growth of the WT strain but to different extents at the concentrations used. The strains sSD003 and sSD019 in one lineage were significantly more tolerant than the wild-type strain to 3% *n*-propanol, growing ~ three- to fourfold faster (*P *< 0.01). Interestingly, sSD021 in the other lineage was significantly less tolerant to *n*-propanol (*P *< 0.05). sSD003 was also more tolerant to 1.2% *n*-butanol (1.3-fold), whereas sSD021 was less tolerant (*P *< 0.001). The more evolved strains, sSD019 and sSD021, were more tolerant to 1.2% isobutanol, growing ~ 1.4-fold faster than the parental strain (*P *< 0.05). Moreover, the evolved strains showed improved tolerance to 0.6% amyl alcohol growing ~ 2 to 3-fold faster, and sSD019 and sSD021 had improved tolerance to 0.4% isoamyl alcohol growing about 20% faster (*P *< 0.05). A pattern of improved tolerance of the evolved strains similar to that seen in *n*-hexanol (Fig. 1), namely sSD019 ≅ sSD021 > sSD003 > sSD006, was observed with the longer-chained alcohols 0.5% *n*-pentanol, 0.05% *n*-heptanol, and 0.05% 3-octanol.

**Table 2:** Medium-chain alcohol tolerance of evolved strains

Strains	*µ*_avg_ (h^−1^)*								
	Alcohol (%, v/v)								
	PrOH (3)^a^	BuOH (1.2)^a^	iBuOH (1.2)	AOH (0.6)	iAOH (0.4)	PtOH (0.6)^a^	HxOH (0.15)^a^	HpOH (0.05)^a^	OcOH (0.05)^a^
WT	0.042 ± 0.004	0.144 ± 0.014	0.195 ± 0.032	0.044 ± 0.013	0.264 ± 0.027	0 ± 0.010	0.043 ± 0.018	0 ± 0.009	0 ± 0.008
sSD003	0.172 ± 0.030^#^	0.170 ± 0.005*	0.202 ± 0.020	0.096 ± 0.026*	0.294 ± 0.023	0.133 ± 0.034^#^	0.252 ± 0.038^†^	0.051 ± 0.026*	0.092 ± 0.009^†^
sSD006	0.038 ± 0.004	0.134 ± 0.021	0.211 ± 0.027	0.071 ± 0.024	0.285 ± 0.024	0.145 ± 0.103	0.115 ± 0.034^†^	0 ± 0.012	0.033 ± 0.021*
sSD019	0.129 ± 0.022^#^	0.140 ± 0.032	0.267 ± 0.017*	0.128 ± 0.019^#^	0.318 ± 0.006*	0.161 ± 0.090*	0.291 ± 0.015^†^	0.290 ± 0.028^†^	0.274 ± 0.063*
sSD021	0.029 ± 0.042*	0.063 ± 0.001^†^	0.264 ± 0.022*	0.139 ± 0.028^#^	0.311 ± 0.017	0.260 ± 0.072^#^	0.217 ± 0.058^†^	0.247 ± 0.007^†^	0.323 ± 0.020^†^

^a^Differences in growth between strains in these alcohols was only apparent at time intervals beyond the 2–10-h period used to calculate *µ*_avg_ for iBuOH, AOH, iAOH, and HxOH. The *µ*_avg_ in these alcohols was calculated for time periods when the cells were in the exponential growth phase. At these longer time interval, in tightly capped tubes, growth may not have been under fully aerobic conditions. Representative growth curves for these alcohols are given in Additional file 1: Figure S2 (PtOH) and Additional file 1: Figure S3 (HpOH)

* Values are mean ± standard deviation evaluated from three biological replicates for all samples except HxOH, for which there were nine biological replicates for WT, seven for sSD003, and four each for the other three strains. Statistical significance was determined by an unpaired two-tailed Student’s *t* test; significant differences from the WT are indicated as follows: ^†^ *P* ≤ 0.001; ^#^ *P* ≤ 0.01; * *P* ≤ 0.05

### Identification of mutations affecting *n*-hexanol tolerance by whole-genome sequencing

To identify genetic targets that can be exploited to alleviate medium-chain alcohol toxicity in *S. cerevisiae*, genomic DNA was isolated from the evolved strains and sequenced to obtain the whole-genome. Mutations were identified by comparison of each sequenced genome to that of the parental strain, which was also sequenced. All of the mutations for each strain are listed in Table 3. Strikingly, *n*-hexanol-tolerant strains in both evolutionary lineages had mutations in translation initiation proteins. These proteins were Gcd1p, the γ subunit of the translation initiation factor eIF2B, Gcd7p, the β subunit of eIF2B, and Sui2p, the α subunit of the translation initiation factor eIF2 (Table 4). The strains sSD003 and sSD019 in one lineage had a D85E mutation in Gcd1p (here designated as the *gcd1*-*1* allele), whereas sSD006 and sSD021 in the other lineage had an R56C mutation in Gcd7p (*gcd7*-*2*). Strain sSD021 had a second translation initiation protein mutation D77Y in Sui2p (*sui2*-*2*). Also of interest is the observation that in the more evolved strains in the two lineages, sSD019 and sSD021, mutations were found in the plasma membrane ABC transporter Pdr5p, one of the major multidrug exporters of *S. cerevisiae*. Strain sSD019 was found to harbor a Q446* mutation (*pdr5*-*1*) that would result in a truncated Pdr5p (tPdr5p^1–446^), and sSD021 had Pdr5p with a G925A mutation (*pdr5*-*2*). Because the other mutations predominantly identified in the more evolved strains differed between the lineages, we assumed that they were passenger mutations that arose from genetic drift, and decided to focus our attention on the translation initiation protein mutations and the role of Pdr5p only.

**Table 3:** Summary of mutations in evolved strains identified by whole-genome resequencing

Strain	Mutation	Gene	Protein function^b^	Mutant allele
sSD003	D85E	*GCD1*	Gamma subunit of the translation initiation factor eIF2B	*gcd1*-*1*
	G338C	*CIT2*	Citrate synthase, peroxisomal isozyme involved in glyoxylate cycle	*cit2*-*1*
sSD006	R56C	*GCD7*	Beta subunit of the translation initiation factor eIF2B	*gcd7*-*2*
sSD019	D85E	*GCD1*	Gamma subunit of the translation initiation factor eIF2B	*gcd1*-*1*
	G338C	*CIT2*	Citrate synthase, peroxisomal isozyme involved in glyoxylate cycle	*cit2*-*1*
	Q446^a^	*PDR5*	Plasma membrane ATP-binding cassette (ABC) transporter	*pdr5*-*1*
	G135V	*UBP13*	Ubiquitin-specific protease	*ubp13*-*1*
	S234C	*LSB6*	Type II phosphatidylinositol 4-kinase	*lsb6*-*1*
	A662G	*NST1*	Protein of unknown function; mediates sensitivity to salt stress	*nst1*-*1*
	Silent	*COX1*	Subunit I of cytochrome c oxidase (Complex IV)	*cox1*-*1*
sSD021	R56C	*GCD7*	Beta subunit of the translation initiation factor eIF2B	*gcd7*-*2*
	D77Y	*SUI2*	Alpha subunit of the translation initiation factor eIF2	*sui2*-*2*
	G925A	*PDR5*	Plasma membrane ATP-binding cassette (ABC) transporter	*pdr5*-*2*
	A529E	*SEY1*	Dynamin-like GTPase that mediates homotypic ER fusion	*sey1*-*2*

^a^Denotes the introduction of an early stop codon

^b^Protein function taken from the *Saccharomyces* genome database website http://www.yeastgenome.org

**Table 4:** Subunits of eIF2 and eIF2B

	Subunit	Protein	Function
eIF2	α	Sui2p	Regulation of eIF2 activity
	β	Sui3p	tRNA binding
	γ	Gcd1 1p	GTP binding
eIF2B	α	Gcn3p	Regulatory subunit; eIF2B regulation
	β	Gcd7p	Regulatory subunit, role in eIF2 binding
	γ	Gcd1p	Catalytic, non-active, role in eIF2 binding
	δ	Gcd2p	Regulatory subunit; eIF2B formation
	ε	Gcd6p	GDP to GTP exchange

**Fig. 2:**
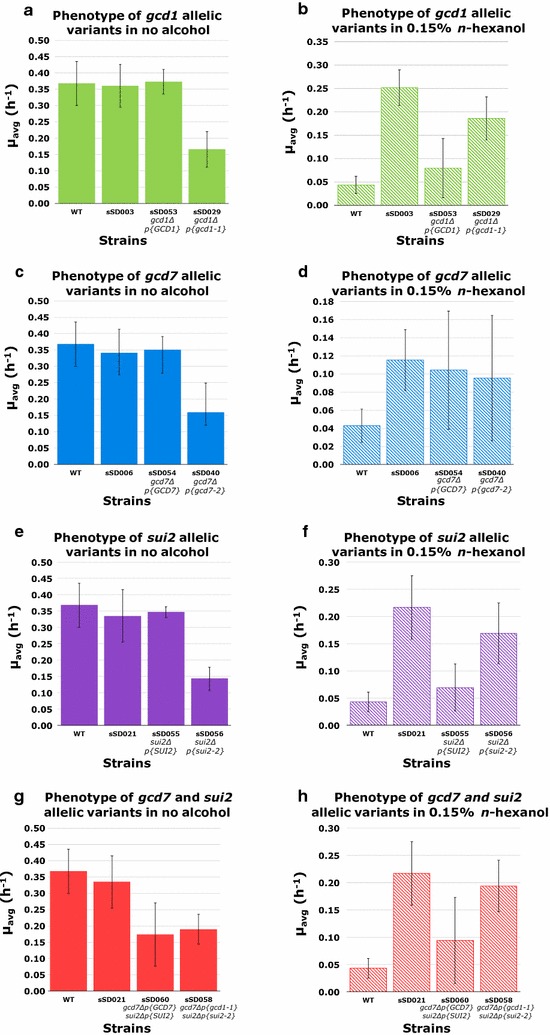
Reverse engineering of *gcd1-1*, *gcd7-2*, and *sui2-2* in *gcd1D*, *gcd7D*, and *sui2D* strains, respectively. For the *gcd1* allelic variants, strains sSD003, sSD053, and sSD029 were grown in 10 ml YPD in the absence (**a**) or presence (**b**) of 0.15% n-hexanol, and their *μ*_*avg*_ values are shown. For the *gcd7* allelic variants, strains sSD006, sSD054, and sSD040 were grown in 10 ml YPD in the absence (**c**) or presence (**d**) of 0.15% n-hexanol, and their *μ*_*avg*_ values are shown. For the sui2 allelic variants, strains sSD021, sSD055, and sSD056 were grown in 10 ml YPD in the absence (**e**) or presence (**f**) of 0.15% nhexanol, and their *μ*_*avg*_ values are shown. For the *gcd1* allelic variants, strains sSD021, sSD060, and sSD058 were grown in 10 ml YPD in the absence (**g**) or presence (**h**) of 0.15% *n*-hexanol, and their *μ*_*avg*_ values are shown. The *μ*^*avg*^ values for WT, sSD003, sSD006 and sSD021 in the absence of alcohol are also given in the text and they are shown here for comparison with relevant reverse-engineered strains. The values for WT, sSD003, sSD006 and sSD021 in the presence of 0.15% *n*-hexanol are the same as in Fig. 1 and are given for comparison. Identical data for WT are shown in multiple plots for comparison and data for strain sSD021 is shown in both **e**, **f** and **g**, **h** for comparison. Values are means ± standard deviation from three independent cultures

To investigate whether the mutations in the translation initiation proteins contribute to the *n*-hexanol tolerant phenotype, we reversed engineered the *gcd1*-*1*, *gcd7*-*2*, and *sui2*-*2* alleles in BY4741. As the *GCD1*, *GCD7*, and *SUI2* genes are essential for viability, the mutant alleles (or the wild-type alleles as controls) were first introduced individually (or in combination as indicated) on low-copy plasmids into BY4741, and then the wild-type genes in the genome of transformed strains corresponding to those on the plasmids were deleted. The *n*-hexanol tolerance of the resulting strains (Table 1) was tested and compared to the parental strain and the relevant evolved strain (Fig. 2). The introduction of *gcd1*-*1*, but not *GCD1*^+^, into BY4741 *gcd1Δ* resulted in a strain (sSD029) that was significantly more tolerant to 0.15% *n*-hexanol than the wild-type strain (*P *< 0.05) (Fig. 2b), though less tolerant than sSD003, the evolved strain with the *gcd1*-*1* allele (*P *< 0.05). These results confirm that *gcd1*-*1* confers *n*-hexanol tolerance in BY4741. Likewise, the strain sSD056 with the *sui2*-*2* allele, but not that carrying the *SUI2*^+^ allele (sSD055), was significantly more tolerant to 0.15% *n*-hexanol than the wild-type strain (*P *< 0.05) (Fig. 2f), and exhibited a tolerance level comparable to sSD021, the evolved strain with the *sui2*-*2*, *gcd7*-*2*, and *pdr5*-*2* alleles. A strain sSD058 carrying both *sui2*-2 and *gcd7*-*2* exhibited an *n*-hexanol tolerance (Fig. 2h) similar to that seen with sSD056 which had only *sui2*-2, suggesting that the tolerance of these strains is largely accounted for by the presence of the *sui2*-*2* allele. These results confirm that *sui2*-*2* confers *n*-hexanol tolerance in BY4741. In contrast to the results with *gcd1*-*1* and *sui2*-*2*, the strain with the reverse-engineered mutant *gcd7*-*2* allele (sSD040), a reconstruction of sSD006, was not significantly more tolerant to 0.15% *n*-hexanol than the wild-type strain (Fig. 2d).

Interestingly, although plasmid-borne *GCD1*^+^ fully complemented the *gcd1Δ* in BY4741 in the absence of alcohol, as evidenced by strain sSD053 having the same growth rate as wild-type BY4741 (Fig. 2a), plasmid-borne *gcd1*-*1* did not [sSD029 grew ~ 2-fold slower than WT in the absence of alcohol (*P *< 0.05)], suggesting that the expression level of *gcd1*-*1* achieved with the plasmid may be lower than that from the chromosome in sSD003. This may account for the failure of plasmid-borne *gcd1*-*1* to fully restore the degree of *n*-hexanol tolerance seen with sSD003. A lack of full complementation of gene deletions was also observed with the other mutant alleles [*gcd7*-*2* (Fig. 2c), *sui2*-*2* (Fig. 2e), and *gcd7*-*2* and *sui2*-*2* (Fig. 2g)]. Moreover, in strain sSD060 (Fig. 2g), full complementation was not achieved with plasmid-borne *GCD7*^+^ and *SUI2*^+^, perhaps reflecting the additional metabolic burden of carrying and replicating two plasmids.

Unpublished work reported in a patent by Tina K. Van Dyk (Butamax^Tm^ Advanced Biofuels Llc) [35] demonstrated that the haploid strain BY4741 ∆pdr5 (*MATa his3∆1 leu2∆0 met15∆0 ura3∆0 pdr5∆::kanMX4*) [36] showed improved tolerance to isobutanol as compared to the parental strain (eightfold increase in 24 h biomass yield in the presence of 1% (w/v) isobutanol). A homozygous diploid ∆pdr5 strain showed improvements in tolerance to *n*-butanol [1.4-fold increase in 24 h biomass yield in the presence of 0.625% (w/v) *n*-butanol]. Pdr5p is known to have a high basal ATPase activity and to be among the major consumers of cellular energy [37], and thus the improvements in isobutanol and butanol tolerance observed for the ∆pdr5 strains likely reflects improved fitness due to loss of the protein. The Q446* mutation in Pdr5p encoding a truncated version of the protein (tPdr5p^1–446^) would certainly abolish transporter activity as only the N-terminal nucleotide-binding domain and not the two transmembrane domains would be expressed [37]. To investigate whether the loss of Pdr5p could make a contribution to the *n*-hexanol tolerance phenotype, we compared the *n*-hexanol tolerance of BY4741 ∆pdr5 with the WT strain. Surprisingly, the ∆pdr5 strain did not show improved *n*-hexanol tolerance [Additional file 1: Figure S4; 0.055 ± 0.003 h^−1^ (*n* = 3) and 0.057 ± 0.005 h^−1^ (*n* = 3), respectively]. This result does not rule out the possibility that the evolved tPdr5p^1–446^ strain, sSD019, (and/or the sSD021 strain with the G925A mutation in Pdr5p) has a gain-of-function mutation that only becomes important when *n*-hexanol toxicity-alleviating mutations are already present in translation initiation proteins.

### Mutations in Gcd1p and Sui2p relieve inhibition of translational activity by higher alcohols

**Fig. 3:**
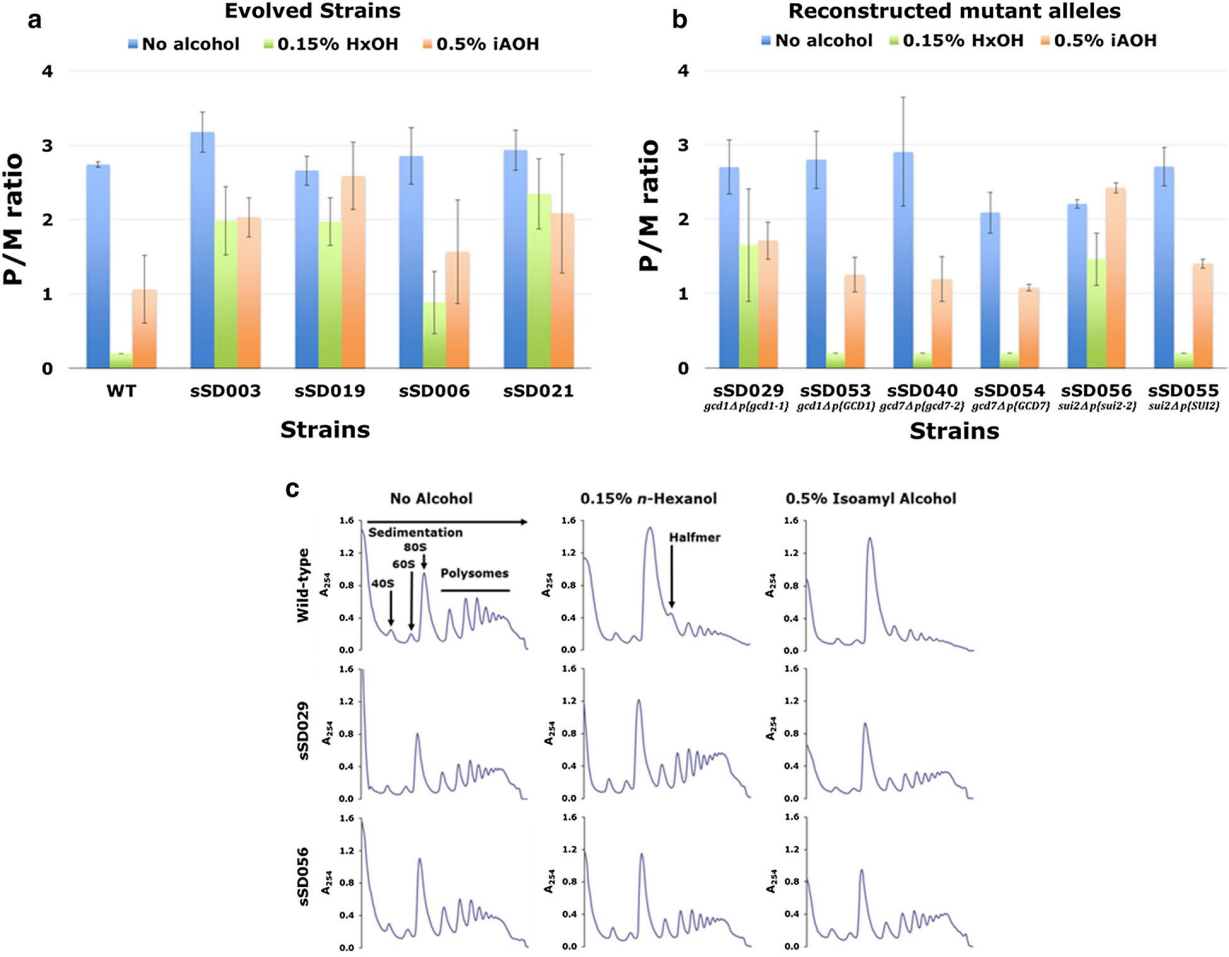
Mutations in Gcd1p and Sui2p relieve inhibition of translation initiation by *n*-hexanol and isoamyl alcohol. All strains were grown in YPD to an OD_600_ of ~ 1 AU. *n*-Hexanol (0.15%) or isoamyl alcohol (0.5%) were then added and the cultures incubated for a further 30 min. Polysomes were analyzed as described in “Methods”. The P/M ratio was determined for **a** WT and evolved strains, and **b** strains with the reconstructed mutant alleles (and wild-type genes as controls). Values are mean ± standard deviation from three independent cultures. **c** Representative polysome profiles for WT, and the reconstructed *gcd1*-*1* (sSD029) and *sui2*-*2* (sSD056) strains. The 40S (small ribosomal unit), 60S (large ribosomal unit), 80S (monosome), halfmer and polysome peaks are labeled

To probe the mechanism by which mutations in Gcd1p and Sui2p cause improved *n*-hexanol tolerance of yeast cells, we examined the translational activity of cells in the presence and absence of *n*-hexanol by polysome profiling. Translational activity is usually expressed as the polysome-to-monosome (P/M) ratio, which, in principle, decreases when translation initiation is defective and increases with elongation defects. In the former case, there is a reduction in the number of ribosomes translating a given mRNA, and hence a reduction in the abundance of polysomes together with a concomitant increase in the amount of free 80S ribosomes [34]. Figure 3a, c shows that the P/M ratio for wild-type cells is dramatically reduced following a 30-min incubation with 0.15% *n*-hexanol (~ 14-fold) (*P *< 0.05). Consistent with the hypothesis that translation initiation is inhibited in this context, we also observe the accumulation of a type of polysome referred to as a halfmer (Fig. 3c). A halfmer results when a 40S complex scans to the start codon of an mRNA that also has an 80S ribosome elongating in the open reading frame, and then there is a defect in 60S ribosomal subunit joining [38]. Isoamyl alcohol also significantly inhibited translation initiation in wild-type cells (> twofold) (*P *< 0.05). In the evolved strains sSD003 and sSD019 from one evolutionary lineage, translational activity was significantly greater than that of the wild-type strain in the presence of both *n*-hexanol and isoamyl alcohol (*P *< 0.05) (Fig. 3a). For the evolved strains sSD006 and sSD021 from the other lineage, significantly greater translational activity was only observed in *n*-hexanol (*P *< 0.05) (Fig. 3a).

Reconstruction of the *gcd1*-*1* allele from sSD003 and sSD019 in BY4741 (sSD029) relieved the inhibition of translational activity observed with *n*-hexanol and isoamyl alcohol in a control strain (sSD053) carrying *GCD1*^+^ (Fig. 3b, c) (*P *< 0.05). Similarly, reverse-engineering of the *sui2*-*2* allele from sSD021 in BY4741 (sSD056) relieved the inhibition of translation initiation observed in sSD055 carrying *SUI2*^+^ (Fig. 3b, c) (*P *< 0.05). In contrast to these results, the introduction of the *gcd7*-*2* allele had no effect on the inhibition of translational activity observed with *n*-hexanol and isoamyl alcohol (Fig. 3b). Taken together, these results suggest that the D85E mutation in Gcd1p and the D77Y mutation in Sui2p relieve the inhibition of translation initiation caused by *n*-hexanol and isoamyl alcohol.

### Amino-acid substitutions of D85 of Gcd1p and D77 of Sui2p modulate *n*-hexanol tolerance

**Fig. 4:**
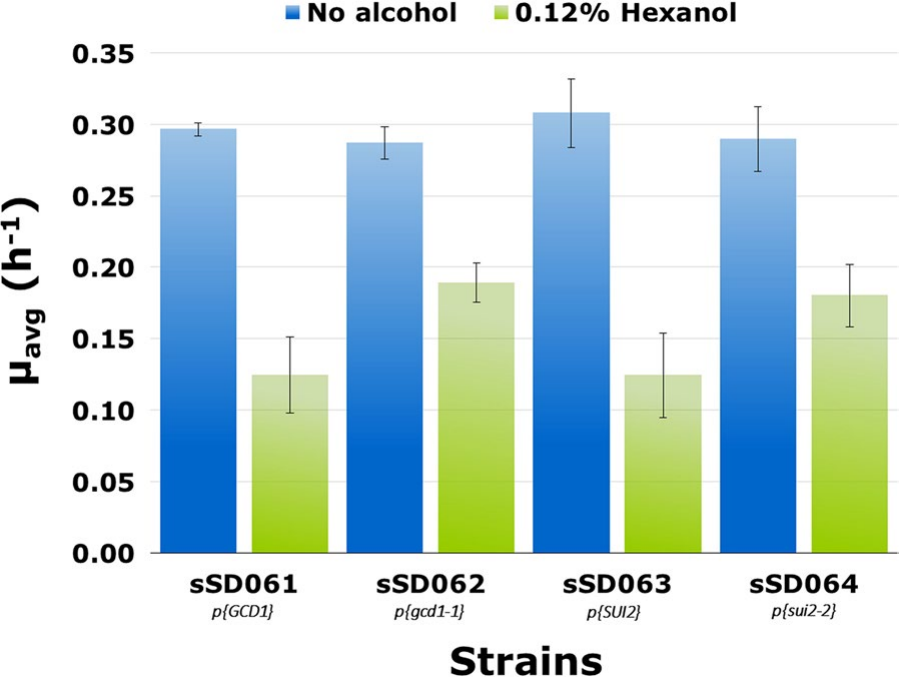
The *n*-hexanol tolerance phenotype conferred by the *gcd1*-*1* and *sui2*-*2* alleles is dominant in BY4741. Strains were grown in 10 ml SD medium lacking uracil in the presence and absence 0.12% *n*-hexanol. Values are mean ± standard deviation from three independent cultures

**Fig. 5:**
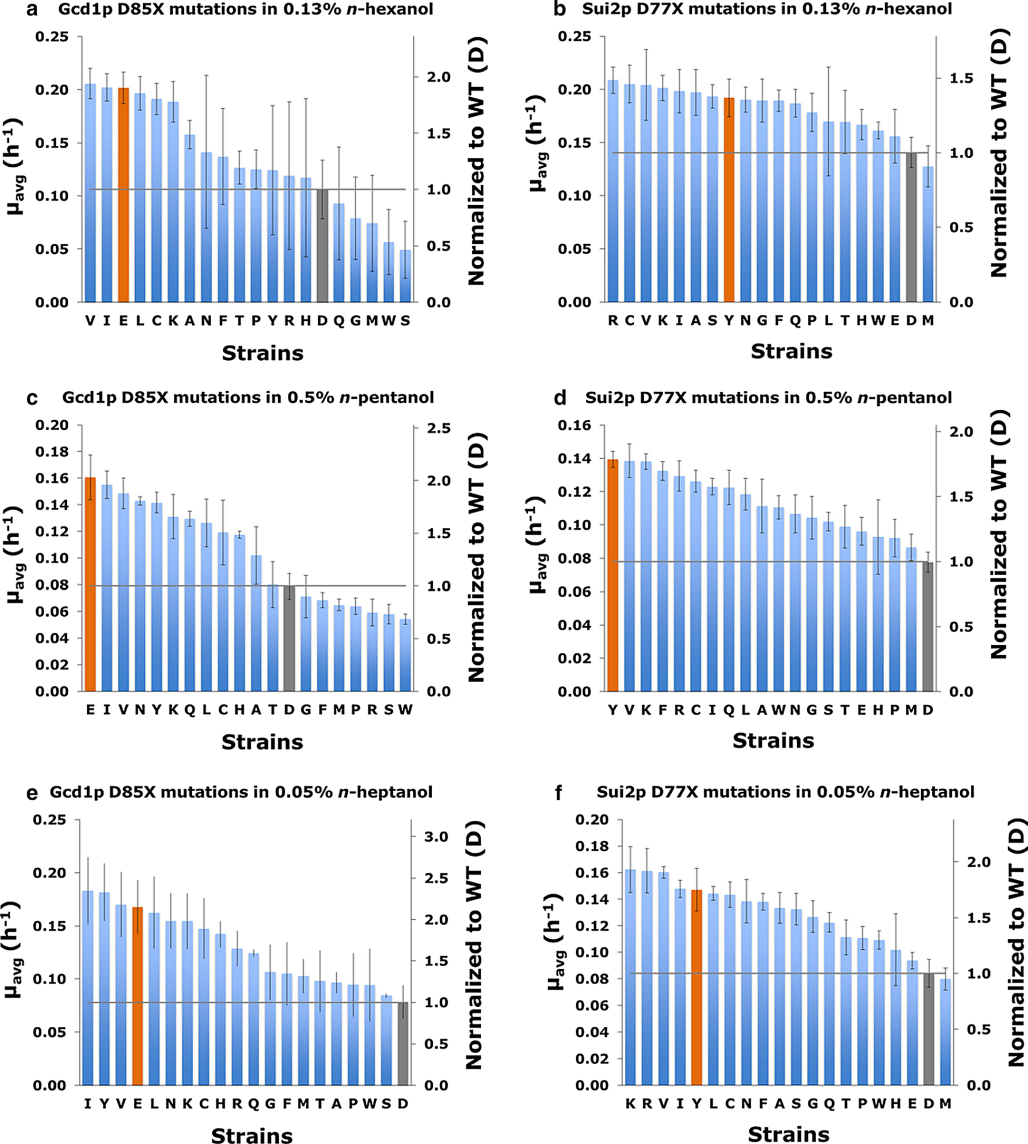
Amino acid substitutions of D85 of Gcd1p and D77 of Sui2p modulate medium-chain alcohol tolerance. Plasmids for the expression of mutant Gcd1p and Sui2p (Additional file 1: Table S2) were transformed into wild-type BY4741. Overnight cultures of strains were diluted to an OD_600_ of 0.15 AU in 10 ml SD medium lacking uracil and containing either 0.13% *n*-hexanol (**a**, **b**), 0.5% *n*-pentanol (**c**, **d**), or 0.05% *n*-heptanol (**e**, **f**). Strains harboring the plasmids expressing Gcd1p mutants are shown in **a**, **c**, and **e**, while strains harboring the plasmids expressing Sui2p mutants are shown in **b**, **d**, and **f**. The μavg was calculated from growth curves for the time period between 4 and 12 h. The grey bar indicates the WT protein, the orange bar, the substitutions identified in the evolved strains. X represents the substituted amino-acid. Values are means ± standard deviation from three independent cultures

Importantly, we found that when the mutant alleles *gcd1*-*1* and *sui2*-*2* were individually introduced into cells containing a chromosomal wild-type copy of the respective gene, the *n*-hexanol tolerance phenotype was still observed (Fig. 4). We exploited this fact to investigate the effect of all possible amino-acid substitutions at positions D85 in Gcd1p and D77 Sui2p D77 on *n*-hexanol, *n*-pentanol, and *n*-heptanol tolerance. We reasoned that the evolved strains give us insight into the important residues for tolerance, but all amino acids may not have been sampled during the ALE. All of the remaining 18 naturally occurring amino acids were substituted at the desired site in each protein. The growth of the resulting strains in the presence of 0.13% *n*-hexanol, 0.5% *n*-pentanol, and 0.05% *n*-heptanol is shown in Fig. 5. The average specific growth rate (*µ*_avg_) values for the Gcd1p mutants in 0.13% *n*-hexanol varied over an ~ fourfold range (0.21–0.05 h^−1^), both above, and surprisingly, below the value obtained for the wild-type protein (0.11 h^−1^) (Fig. 5a). A similar pattern was observed in 0.5% *n*-pentanol (~ 3-fold range of *µ*_avg_ from 0.16 to 0.05 h^−1^ with a wild-type value of 0.08 h^−1^) (Fig. 5c). The growth rates which were significantly lower than that for the wild-type protein (*P *< 0.05) may suggest that the expression of these Gcd1p mutants was itself toxic to yeast cells. Growth rates for the Gcd1p mutants in 0.05% *n*-heptanol varied over a ~ 2-fold range (0.18–0.08 h^−1^) with the wild-type protein having the lowest value (Fig. 5e). Interestingly, in all three alcohols, the D85E substitution, which was identified in the evolved strains sSD003 and sSD019, resulted in the highest level of tolerance; no other substitution led to significantly greater alcohol tolerance. The amino-acid substitutions in Gcd1p that confer increased tolerance to alcohols do not share any chemical or structural property that might readily account for why they do so. Substitutions of aliphatic, hydrophobic amino-acids (leucine, isoleucine, and valine) produced mutants that were at the high end of the tolerance spectrum, but so did the introduction of the polar amino-acid lysine, which resulted in a change in the charge at this position.

Tolerance levels for the Sui2p mutants in 0.13% *n*-hexanol, 0.5% *n*-pentanol, and 0.05% *n*-heptanol varied over a 1.6-fold range (0.21–0.13 h^−1^), 1.8-fold range (0.14–0.08 h^−1^), and twofold range (0.16–0.08 h^−1^), respectively, and in each case, the wild-type protein had the lowest, or second lowest tolerance (Fig. 5b, d, f). Once again, in all three alcohols, the D77Y substitution, which was identified in the evolved strain sSD021, resulted in the highest level of tolerance with no other substitution giving rise to significantly greater alcohol tolerance. As for the Gcd1p mutants, no single amino-acid property readily accounts for the improved alcohol tolerance observed with the Sui2p mutants. In fact, nearly all substitutions at D77 in Sui2p resulted in significantly improved alcohol tolerance.

## Discussion

A significant challenge in the development of cost-competitive production processes for advanced higher alcohol biofuels in *S. cerevisiae* is that these compounds are often highly toxic to yeast cells. This problem provided the motivation for the current work in which we sought to identify genetic targets that can be exploited in tolerance engineering and strain development for biofuel production. *S. cerevisiae* strains adapted for growth in *n*-hexanol concentrations as high as 0.2% were isolated through ALE. The evolved strains showed increased tolerance to *n*-hexanol which was most pronounced in the concentration range 0.15–0.2% (Fig. 1b) where the parental strain was unable to grow. Although difficult to evaluate the significance of these tolerance increases from the point of view of the contribution they might make to *n*-hexanol production, a recent study of *n*-butanol production in *S. cerevisiae* demonstrated that a strain that was more resistant to the toxic effects of butanol produced significantly more of it [6]. Future studies will have to establish whether the increased tolerance of yeast cells to *n*-hexanol, and other medium-chain alcohols, afforded by these mutations leads to increased titers of target bio-alcohols in production strains.

Remarkably, in both of the two independent evolutionary lineages of *n*-hexanol-tolerant *S. cerevisiae* established in the current study, mutations in translation initiation proteins were identified. One mechanism for the toxicity of the fusel alcohols (*n*-butanol, isobutanol, 2-methylbutan-2-ol, and isoamyl alcohol) in *S. cerevisiae* involves inhibition of translation initiation due to perturbation in eIF2B activity that lead to reduced levels of the eIF2-GTP Met-tRNA^i^ ternary complex [14, 15]. Moreover, fusel alcohol-dependent translational inhibition is modulated by mutations in all five eIF2B subunits (subunits of the catalytic subcomplex: γ (Gcd1p) [14, 15] and ε (Gcd6p) [15, 39]; subunits of the regulatory subcomplex; α (Gcn3p) [15], β (Gcd7p) [39], and δ (Gcd2p) [14]). In one of our lineages, a D85E mutation in Gcd1p occurred during the first 30-day round of adaptive evolution. Reverse-engineering of this mutation demonstrated that it conferred *n*-hexanol tolerance to BY4741 (Fig. 2a, b). Polysome profiling revealed that *n*-hexanol does indeed inhibit translation initiation as evidenced by a dramatic decrease in the P/M ratio (as compared to a no alcohol control) and the appearance of halfmers (Fig. 3a, c), and that reconstruction of the D85E Gcd1p mutation in BY4741 significantly attenuated inhibition of translational activity (Fig. 3b, c). These results suggest that *n*-hexanol likely shares the same eIF2B-mediated mechanism of translational inhibition as the fusel alcohols. Remarkably, examination of all possible amino-acid substitutions at position D85 in Gcd1p revealed that the evolved mutation D85E resulted in the highest possible level of tolerance to medium-chain alcohols (Fig. 5a, c, e). In contrast to the D85E eIF2Bγ mutation identified here, other mutations in this subunit (*gcd1*-P180S, *gcd1*-C483W, and *gcd1*-*101*) were found to increase sensitivity to alcohol [14].

In our second evolutionary lineage, mutations occurred in two other translation initiation proteins, and the more significant of the two, a D77Y mutation in Sui2p, the α subunit of the translation initiation factor eIF2 (eIF2α), occurred during the second 30-day round of ALE. Reverse-engineering of this mutation demonstrated that it too conferred *n*-hexanol tolerance to BY4741 (Fig. 2e, f) by relieving inhibition of translation initiation (Fig. 3b, c). And as was found for our eIF2Bγ mutation, site-saturation mutagenesis experiments showed that the evolved D77Y mutation in Sui2p resulted in the highest possible level of tolerance to medium-chain alcohols (Fig. 5b, d, f), highlighting the effectiveness of ALE for tolerance engineering. To our knowledge, this is the first report of a mutation in eIF2 that modulates alcohol-dependent translational inhibition, and the first to suggest eIF2α as a genetic target for tolerance engineering to medium-chain alcohols in *S. cerevisiae*.

The remaining translation initiation protein mutation in our second lineage was R56C in eIF2Bβ, which occurred in the first 30-day round of ALE. Although the evolved strain sSD006, which contains this mutation as the sole mutation detected, exhibited improved tolerance to 0.15% *n*-hexanol (Fig. 1a) [and 0.05% 3-octanol (Table 2)] and had significantly higher translational activity in the presence of *n*-hexanol than the WT strain (Fig. 3a), we were unable to show that the mutation, when reconstructed in BY4741, actually conferred *n*-hexanol tolerance (Fig. 2c, d) or affected *n*-hexanol-dependent inhibition of translation (Fig. 3b). This may have been due to the fact, noted earlier, that full complementation of BY4741 *gcd7Δ* by plasmid-borne *gcd7*-*2* was most likely not achieved. If too little of the mutant Gcd7p was expressed in the reconstructed strain, this may itself have led to lower eIF2B activity. Two mutations in eIF2Bβ, *gcd7*-*201* and *gcd7*-V341D, make growth more sensitive to *n*-butanol [39], supporting the notion that the eIF2Bβ^R56C^ mutant identified here may play a role in *n*-hexanol tolerance.

The evolved strains reported in this paper (particularly, sSD003, sSD019, and sSD021) showed improved tolerance to a wide range of alcohols (Table 2), suggesting that the mutations in the translation initiation proteins identified here (particularly D85E in Gcd1p and D77Y in Sui2p) may play a role in alleviating the impact of these alcohols on the translational apparatus, and may be useful in work directed at producing these alcohols in *S. cerevisiae*. An interesting observation in these data was that the strain sSD003 containing the D85E mutation in Gcd1p and the strain sSD021 with the D77Y mutation in Sui2p behaved differently with respect to growth in *n*-propanol and butanol (Table 2). sSD003 showed improved tolerance to these alcohols whereas sSD021 was more sensitive to them. This result suggests that these mutations may prove useful in probing the mechanism by which these alcohols interact with eIF2/eIF2B to alter their activity.

In the absence of any information on where fusel alcohols and other medium-chain alcohols bind to eIF2B, and perhaps also to eIF2, the specific roles of the translation initiation protein mutations identified here in alleviating alcohol-dependent translational inhibition remain unresolved and will require further work. Nevertheless, the observation that a mutation in eIF2α, in addition to those in eIF2B, can rescue alcohol-dependent translational inhibition, suggests that alcohols may perturb eIF2/eIF2B activity by binding at the interface of the proteins, and in so doing, alter their interaction. Recently, the crystal structure of the *Schizosaccharomyces pombe* eIF2B was determined [40], and studies aimed at experimentally mapping the interaction surfaces between eIF2 and eIF2B were performed [40, 41]. The locations of the homologous residues in the *S. pombe* eIF2Bγ (E81, P156, and C411) corresponding to those found to modulate alcohol sensitivity in the *S. cerevisiae* eIF2Bγ (D85, P180, C483, respectively) are shown in the *S. pombe* eIF2B structure in Additional file 1: Figure S5. Also shown in the red hatched box are the location of surfaces of eIF2Bγ and eIF2Bε found to make contacts with eIF2γ, the eIF2 subunit to which GTP is bound [40, 41]. E81 and P156 are located in the vicinity of this interaction surface, whereas C411 is further away. The homologous residue corresponding to R56 in the *S. pombe* eIF2Bβ is R47 which is located on the surface of the subunit, but outside of the areas shown to be involved in interactions with eIF2γ (red hatched box) and eIF2α (orange hatched box). The residue corresponding to V341 in eIF2Bβ, I358, is not surface located.

The structure of the N-terminal region of the *S. cerevisiae* eIF2α has also been determined by X-ray crystallography [42]. Mutational analysis of eIF2α has identified two non-contiguous segments comprising residues E49 and R88 (that form a salt-bridge), in close proximity to the phosphorylation site, S51, and residues K79 and G80, located 30 or more residues away from S51, that may be involved in binding to eIF2B [42, 43] (Additional file 1: Figure S6). Phosphorylation of S51 of eIF2α enhances its interaction with eIF2B. The alcohol-sensitive D77 residue (D76 in the structure that lacks M1) is well placed to influence eIF2α binding to eIF2B.

## Conclusions

Analysis of *n*-hexanol-tolerant *S. cerevisiae* strains isolated by ALE revealed that a mechanism of *n*-hexanol toxicity in yeast is inhibition of translation initiation. Mutations in three translation initiation protein genes, *GCD1*, *GCD7*, and *SUI2*, significantly increased tolerance to *n*-hexanol (and a range of other medium-chain alcohols), and can be exploited in future metabolic engineering efforts for the production of *n*-hexanol and other medium-chain alcohols.

## Additional file

**Additional file 1: Figure S1.** Representative growth curves for evolved strains in 0.15% *n*-hexanol. Strains were grown in 10 ml YPD + 0.15% *n*-hexanol in capped 50 ml culture tubes. **Figure S2.** Growth curves for evolved strains in 0.5% *n*-pentanol. **Figure S3.** Growth curves for evolved strains in 0.05% *n*-heptanol. **Figure S4.** Growth curves to examine the impact of Pdr5 in 0.15% *n*-hexanol. **Figure S5.** Structure of *S. pombe* eIF2B with the positions of alcohol-sensitive *S. cerevisiae* eIF2Bγ and eIF2Bβ mutations mapped. The subunit arrangement depicted is of the α_2_β_2_δ_2_ hexameric regulatory subcomplex bound to two γε dimeric catalytic subcomplexes on its opposite sides [40]. The α-, β-, γ-, δ- and ε-subunits are colored blue, cyan, orange, green and pink, respectively. The positions of mapped alcohol-sensitive *S. cerevisiae* eIF2Bγ and eIF2Bβ mutations are indicated on the eIF2Bγε subcomplex on the left only. The location of the interfaces for eIF2γ and eIF2α binding to eIF2B are indicated by the red and orange hatched boxes, respectively [40, 41]. **Figure S6.** Structure of *S. cerevisiae* eIF2α (residues 1–175) [42] indicating the phosphorylation site, S51, the alcohol-sensitive D77 (D76 in structure that lacks M1), and residues that have been shown to be important for interaction with eIF2B (E49, K79, G80, and R88) [42, 43]. **Table S1.** List of primers used in this study. **Table S2.** List of plasmids for expression of Gcd1p and Sui2p mutants.
