# Supplementary material for: Evolutionary engineering improves tolerance for medium-chain alcohols in Saccharomyces cerevisiae
Source: Biotechnol Biofuels. 2018 Apr 2;11:90. doi: 10.1186/s13068-018-1089-9 (PMC5880003; doi:10.1186/s13068-018-1089-9)
Supplement: Supplementary file 1 — Additional file 1: Figure S1. Representative growth curves for evolved strains in 0.15% n-hexanol. Strains were grown in 10 ml YPD + 0.15% n-hexanol in capped 50 ml culture tubes. Figure S2. Growth curves for evolved strains in 0.5% n-pentanol. Figure S3. Growth curves for evolved strains in 0.05% n-heptanol. Figure S4. Growth curves to examine the impact of Pdr5 in 0.15% n-hexanol. Figure S5. Structure of S. pombe eIF2B with the positions of alcohol-sensitive S. cerevisiae eIF2Bγ and eIF2Bβ mutations mapped. The subunit arrangement depicted is of the α2β2δ2 hexameric regulatory subcomplex bound to two γε dimeric catalytic subcomplexes on its opposite sides [40]. The α-, β-, γ-, δ- and ε-subunits are colored blue, cyan, orange, green and pink, respectively. The positions of mapped alcohol-sensitive S. cerevisiae eIF2Bγ and eIF2Bβ mutations are indicated on the eIF2Bγε subcomplex on the left only. The location of the interfaces for eIF2γ and eIF2α binding to eIF2B are indicated by the red and orange hatched boxes, respectively [40, 41]. Figure S6. Structure of S. cerevisiae eIF2α (residues 1–175) [42] indicating the phosphorylation site, S51, the alcohol-sensitive D77 (D76 in structure that lacks M1), and residues that have been shown to be important for interaction with eIF2B (E49, K79, G80, and R88) [42, 43]. Table S1. List of primers used in this study. Table S2. List of plasmids for expression of Gcd1p and Sui2p mutants. [file 13068_2018_1089_MOESM1_ESM.pdf]

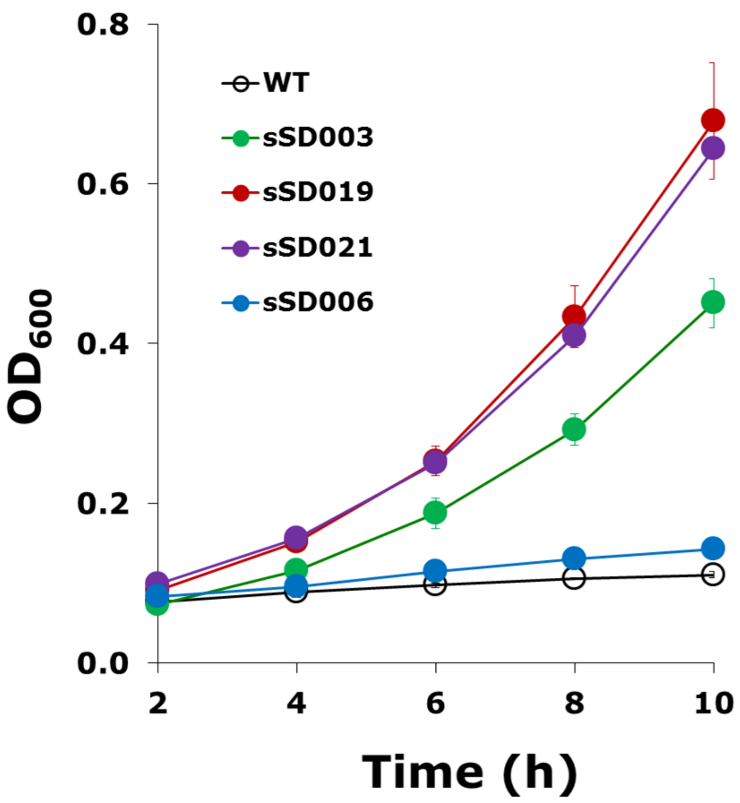

Figure S1. Representative growth curves for evolved strains in 0.15% *n*-hexanol. Strains were grown in 10 ml YPD + 0.15% *n*-hexanol in capped 50 ml culture tubes.

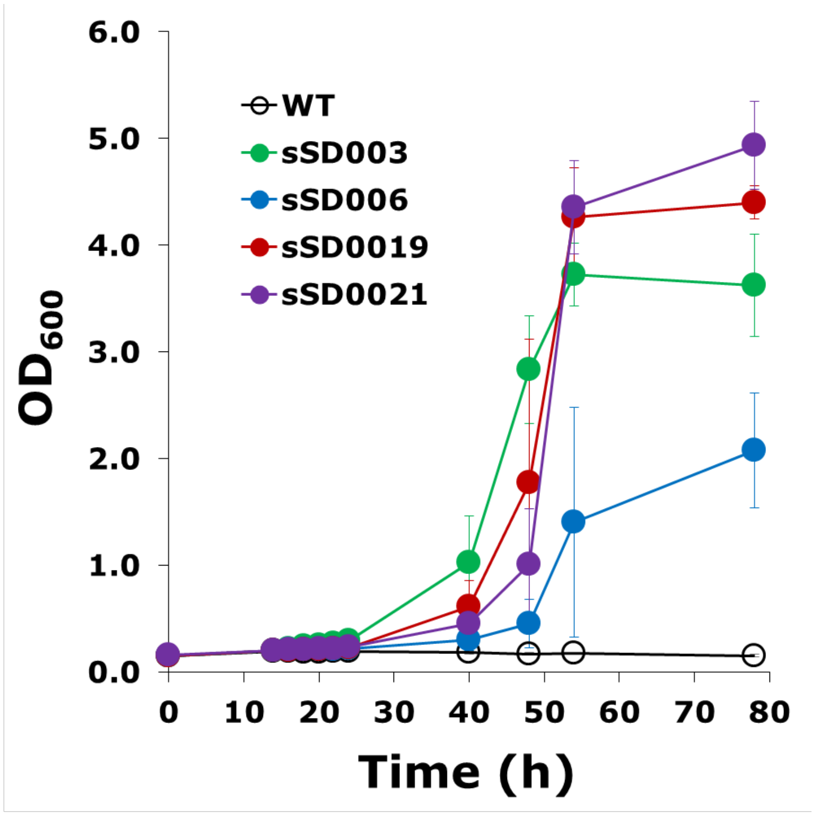

Figure S2. Growth curves for evolved strains in 0.5% *n*-pentanol.

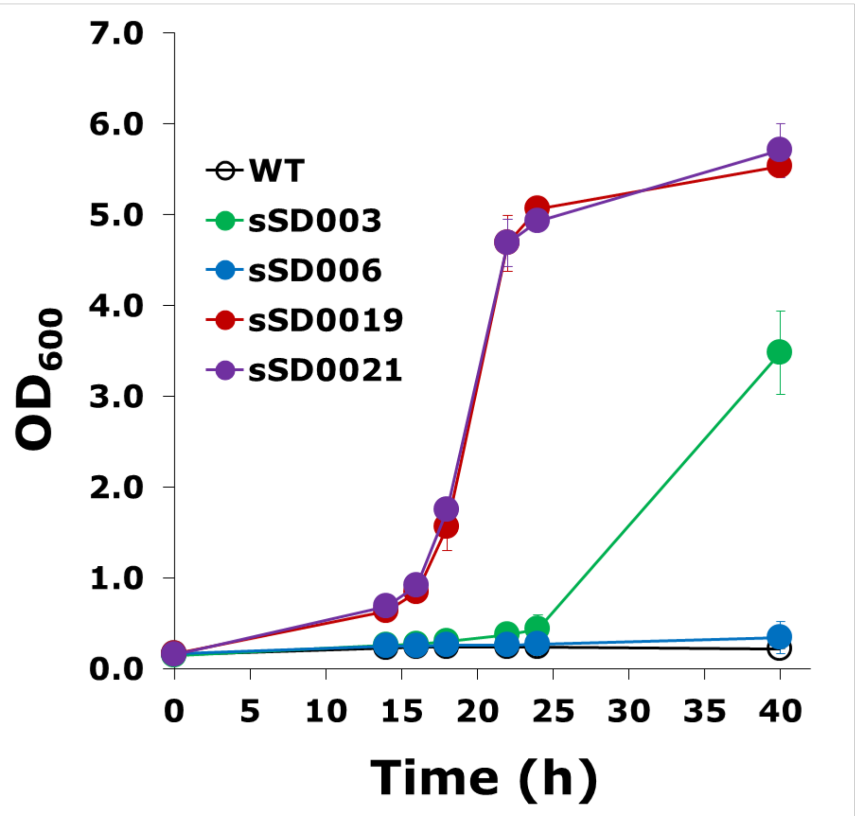

Figure S3. Growth curves for evolved strains in 0.05% *n*-heptanol.

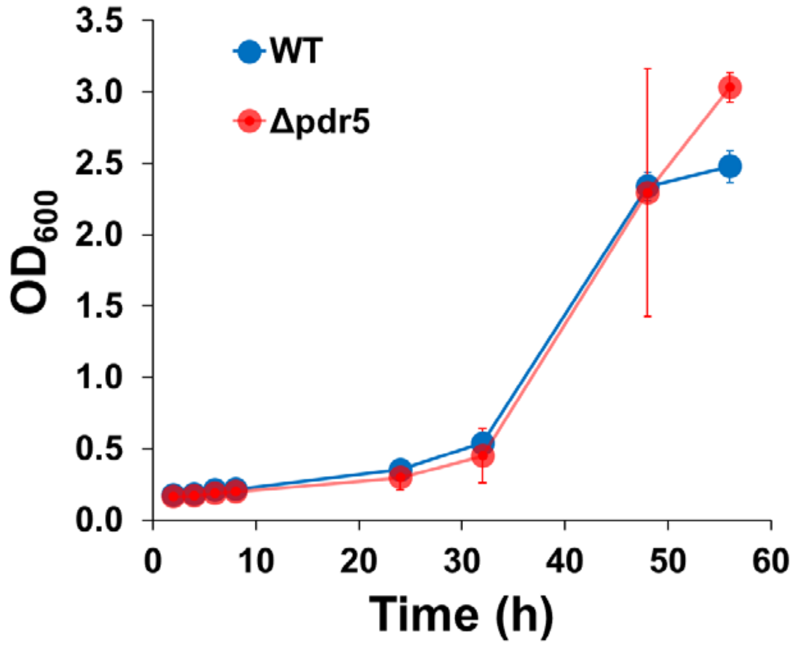

Figure S4. Growth curves to examine the impact of Pdr5 in 0.15% *n*-hexanol.

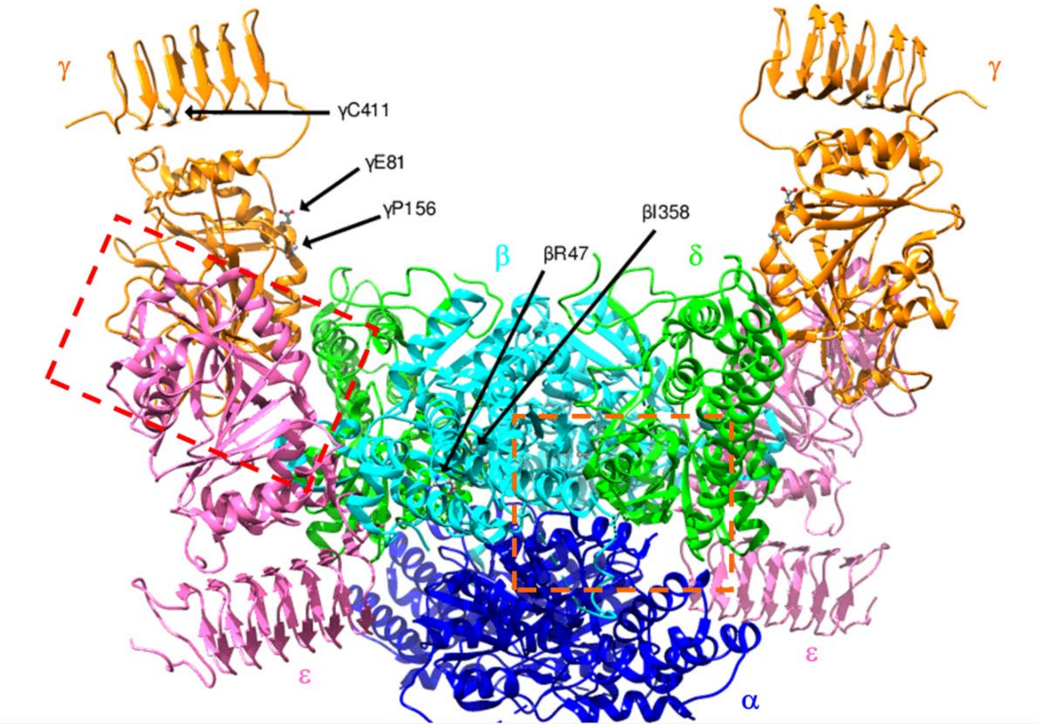

Figure S5. Structure of *S. pombe* eIF2B with the positions of alcohol-sensitive *S. cerevisiae* eIF2Bγ and eIF2Bβ mutations mapped. The subunit arrangement depicted is of the $\alpha_2\beta_2\delta_2$ hexameric regulatory subcomplex bound to two γε dimeric catalytic subcomplexes on its opposite sides [40]. The α-, β-, γ-, δ- and ε-subunits are colored blue, cyan, orange, green and pink, respectively. The positions of mapped alcohol-sensitive *S. cerevisiae* eIF2Bγ and eIF2Bβ mutations are indicated on the eIF2Bγε subcomplex on the left only. The location of the interfaces for eIF2γ and eIF2α binding to eIF2B are indicated by the red and orange hatched boxes, respectively [40, 41].

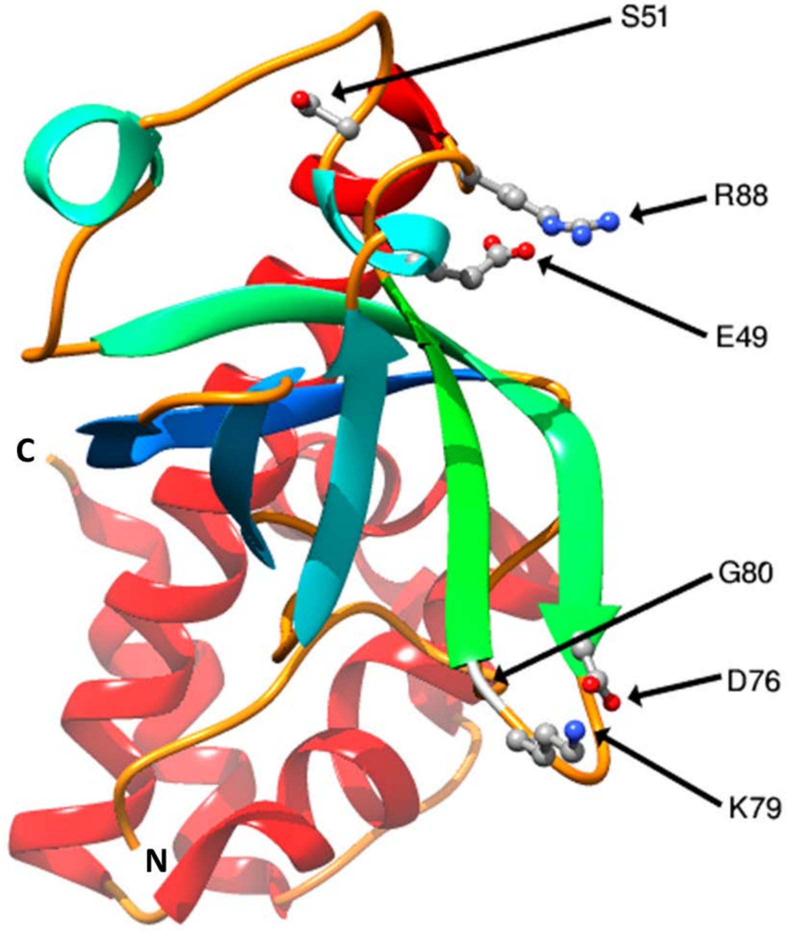

Figure S6. Structure of *S. cerevisiae* eIF2α (residues 1-175) [42] indicating the phosphorylation site, S51, the alcohol-sensitive D77 (D76 in structure that lacks M1), and residues that have been shown to be important for interaction with eIF2B (E49, K79, G80, and R88) [42, 43].

Table S1 List of primers used in this study

Primers	Sequence (From 5’ to 3’)
GCD1-F	GGGCGAATTGGAGCTCACTCGGAATTCAGTCTACTAGTGATCACGGTCATCTCTTATG
GCD1-R	ATCATAAATCATAAGAAATTCGCCTCGAGTTAGGATCCGAAAACATGAATGAAATGTGTA
GCD7-F	GGGCGAATTGGAGCTCACTCGGAATTCAGTCTACTAGTTACAACTTTGAAAAGCCTTTC
GCD7-R	ATCATAAATCATAAGAAATTCGCCTCGAGTTAGGATCCAGTGCAGTATGAATAAAAGACC
SUI2-F	GGGCGAATTGGAGCTCACTCGGAATTCAGTCTACTAGTATTGCGAGATGAAATATAAACC
SUI2-R	ATCATAAATCATAAGAAATTCGCCTCGAGTTAGGATCCAACCATCATAGGAAATACCTTC
GCD1-KanMX-F	ATAAAGGGTCCTGTTCTTCACTCATTCTTAGTTATTAATACGTACGCTGCAGGTCGAC
GCD1-KanMX-R	AAGAGTAAAGGACAAACAATCAAAAGTCTCTGATGACAGTATCGATGAATTCGAGCTCG
GCD7-HIS3-F	CAACACGCCAACTTTCGAACTTTTGCCCAACATAAGTAATTCCGTTTTAAGAGCTTGGTG
GCD7-HIS3-R	TTTTATTTATTTTCATGAGTAATGTACAAAAGACACATCATATGATCCGTCGAGTTCAAG
SUI2-KanMX-F	GTGAGCATTCATCGTTCAGCTCAAAATACGTTTCTTGTCACGTACGCTGCAGGTCGAC
SUI2-KanMX-R	GGAGCCTCATATTATTACAGCATCACTACCTTTTGAGGTAAATCGATGAATTCGAGCTCG
KanMX-diagnostic-F	GCAGTTTCATTTGATGCTCGATG
KanMX-diagnostic-R	GAACCTCAGTGGCAAATCC
HIS3-diagnostic-R	GAGTCATCCGCTAGGTGG
KanMX-sub-SUI2-F	GTCTTTTCTGCTGCCTCACGCACCTTCTATAATACACCAAATACGTACGCTGCAGGTC
KanMX-sub-SUI2-R	TGACACTTGAAAACACCTAGAAAAATTAGGCGCGGCAATGAATCGATGAATTCGAGCTCG
SUI2-library#1-F	TCTTACCCGCAGTCGGAGA
SUI2-library#1-R	CTTTTTCTTTNNNGACACGAAGAACAACGGCGACA
SUI2-library#2-R	GCATGCTCCACTGACAACC
SUI2-library#2-F	CCGTTGTTCTTCGTGTCNNNAAAGAAAAAGGTTATATTGATTTGTCCA
KanMX-sub-GCD1-F	AAAGTTCCCACCGTTGATAGCTCCCCCCCCTATTGTCGTAGTCCGTACGCTGCAGGTC
KanMX-sub-GCD1-R	GTCTCTATTAAAGAGACTGAAGGAATATACATAAGTTTATAATCGATGAATTCGAGCTCG
GCD1-library#1-F	GGTCCTGTTCTTCACTCATTCTTAG
GCD1-library#1-R	GAAATCTGCCTGNNNACACCAATCCAAGACG
GCD1-library#2-F	CGTCTTGGATTGGTGTNNKCAGGCAGATTTC
GCD1-library#2-R	GAGTAAAGGACAAACAATCAAAAGTCTC

The underlined nucleotides correspond to homologous sequences. Double underlined nucleotides correspond to degenerate codons.

Table S2 List of plasmids for expression of Gcd1p and Sui2p mutants

Plasmid	Sui2p mutants	Plasmid	Gcd1p mutants
pSD538	Sui2 D77A	pSD557	Gcd1 D85A
pSD539	Sui2 D77C	pSD558	Gcd1 D85C
pSD540	Sui2 D77E	pSD559	Gcd1 D85F
pSD541	Sui2 D77F	pSD560	Gcd1 D85G
pSD542	Sui2 D77G	pSD561	Gcd1 D85H
pSD543	Sui2 D77H	pSD562	Gcd1 D85I
pSD544	Sui2 D77I	pSD563	Gcd1 D85K
pSD545	Sui2 D77K	pSD564	Gcd1 D85L
pSD546	Sui2 D77L	pSD565	Gcd1 D85M
pSD547	Sui2 D77M	pSD566	Gcd1 D85N
pSD548	Sui2 D77N	pSD567	Gcd1 D85P
pSD549	Sui2 D77P	pSD568	Gcd1 D85Q
pSD550	Sui2 D77Q	pSD569	Gcd1 D85R
pSD551	Sui2 D77R	pSD570	Gcd1 D85S
pSD552	Sui2 D77S	pSD571	Gcd1 D85T
pSD553	Sui2 D77T	pSD572	Gcd1 D85V
pSD554	Sui2 D77V	pSD573	Gcd1 D85W
pSD555	Sui2 D77W	pSD574	Gcd1 D85Y
